# Sex-Specific 25-Hydroxyvitamin D Threshold Concentrations for Functional Outcomes in Older Adults

**DOI:** 10.1093/geroni/igab046.1814

**Published:** 2021-12-17

**Authors:** Michelle Shardell, Jack Guralnik, Eleanor Simonsick, Stephen Kritchevsky, Peggy Cawthon

**Affiliations:** 1 University of Maryland School of Medicine, Baltimore, Maryland, United States; 2 University of Maryland, Baltimore, Baltimore, Maryland, United States; 3 National Instute on Aging/NIH, Baltimore, Maryland, United States; 4 Wake Forest School of Medicine, Winston Salem, North Carolina, United States; 5 California Pacific Medical Center, San Francisco, California, United States

## Abstract

25-Hydroxyvitamin D [25(OH)D] has extra-skeletal effects, but it is not known whether the minimum sufficient serum levels for such targets, like muscle, differ from those for bone health (typically recommended at 20 or 30 ng/dL). Therefore, we derived and validated sex-specific thresholds for serum 25(OH)D predictive of poor physical function using 5 cohorts comprising 16,388 community-dwelling older adults (60.9% women). Using a cohort-stratified random two-thirds sample, we found incident slow gait was best discriminated by 25(OH)D<24.0 versus 25(OH)D>=24.0 ng/mL among women (Relative Risk=1.29; 95% CI 1.10-1.50) and 25(OH)D<21.0 versus 25(OH)D >=21.0 ng/mL among men (RR=1.43; 95% CI 1.01-2.02). Estimates from the remaining one-third validation sample were similar. Empirically identified and validated sex-specific 25(OH)D thresholds from multiple well-characterized cohorts of older adults may yield more biologically meaningful definitions in important sub-populations. Such thresholds may serve as candidate reference concentrations or inform design of vitamin D intervention trials in older adults.

